# SDS Can Be Utilized as an Amyloid Inducer: A Case Study on Diverse Proteins

**DOI:** 10.1371/journal.pone.0029694

**Published:** 2012-01-12

**Authors:** Javed Masood Khan, Atiyatul Qadeer, Sumit Kumar Chaturvedi, Ejaz Ahmad, Syed Arif Abdul Rehman, Samudrala Gourinath, Rizwan Hasan Khan

**Affiliations:** 1 Interdisciplinary Biotechnology Unit, Aligarh Muslim University, Aligarh, India; 2 School of Life Sciences, Jawaharlal Nehru University, New Delhi, India; University of South Florida College of Medicine, United States of America

## Abstract

Sodium dodecyl sulphate (SDS), an anionic surfactant that mimics some characteristics of biological membrane has also been found to induce aggregation in proteins. The present study was carried out on 25 diverse proteins using circular dichroism, fluorescence spectroscopy, dye binding assay and electron microscopy. It was found that an appropriate molar ratio of protein to SDS readily induced amyloid formation in all proteins at a pH below two units of their respective isoelectric points (pI) while no aggregation was observed at a pH above two units of pI. We also observed that electrostatic interactions play a leading role in the induction of amyloid. This study can be used to design or hypothesize a molecule or drug, which may counter act the factor responsible for amyloid formation.

## Introduction

While the majority of the proteins may acquire completely folded and functional conformation under *in-vivo* conditions, some of these undergo misfolding due to several reasons. It still remains unclear whether amyloid development is commenced by native state of proteins, partially or totally unfolded states. The amyloid formation is often linked with genetic mutations that can efficiently destabilize the native state suggesting that a conformational change is the first essential step in amyloidogenesis [1]–[2]. The partially unfolded states of a protein produced under high temperature, high pressure, low pH or moderate concentrations of organic solvents exhibit more propensity to aggregate. Due to several biological and environmental factors, the partially folded intermediates of proteins undergo α/β or β-sheet transition, a characteristic feature of amyloidosis. Amyloid formation is usually a pathogenic process where proteins undergo a conformational change and self-assemble into nonfunctional but toxic fibrils [3]. This process is linked with wide range of human disorders such as Alzheimer's and Creutz-feldt-Jacob disease, senile systemic amyloidosis, type 2 diabetes and dialysis amyloidosis, etc. Amyloid fibrils are also formed in endogenous proteins where they perform normal functions. Hence amyloid formation is not always harmful as evidenced from the simplest to complex organisms [4]–[5]. Sodium dodecyl sulfate (SDS), an anionic surfactant having negatively charged head group and hydrophobic tail, mimics the structure of lipid molecules of biological membranes. SDS is often used as a denaturant that destroys protein's native conformation; it provides an anionic micellar interface that has been shown to accelerate the aggregation of A*β* (1–40) over a limited range of (low SDS) concentrations [6]. Understanding the nature of protein–surfactant interaction is of vital interest and allows us to gain insight into the binding mechanism between the two components and its consequence effect on protein structure and functions in the complex [7]–[8]. Since the conditions required to induce aggregation vary from protein to protein, the objective of this study was to provide a simple legislative to induce protein aggregation or amyloid formation with SDS by choosing appropriate pH conditions based on isoelectric point (pI). The aggregates so formed were studied using various spectroscopic as well as microscopic methods including circular dichroism, turbidity measurements, intrinsic fluorescence, Thioflavin T (ThT) binding and transmission electronic microscopy.

## Materials and Methods

### Materials

Human serum albumin [HSA] (5.67), porcine serum albumin [PSA] (5.89), bovine serum albumin [BSA] (5.65), sheep serum albumin [SSA] (5.63), rabbit serum albumin [RSA] (5.70), ovalbumin [Oval] (4.5), *Mucor javanicus* lipase [M. Java] (5.90), lysozyme [Lyso] (11.2), invertase [Invert] (3.4), hemoglobin [Hb] (7.1), glucose oxidase [GOD] (4.94), Gelatin (4.7), fetuin (5.21), concanavalin A [ConA] (5.43), α-lactalbumin [ALA] (4.4), cobra toxin [Cobra] (7.69), *Candida rugosa* lipase [Cand] (4.7), β-lactoglobulin [BLG] (5.2), bovine liver catalase [BLC] (5.4), α-amylase [Alp Amy] (5.9), conalbumin [CA] (6.69), chymopapain [Chymo] (10.4), rat IgG [RIgG] (7.8), asialofetuin [AFT] (5.1), banana lectin [BL] (6.26) and SDS were purchased from Sigma Chemical Co. (St. Louis, MO, USA). All other reagents used were of analytical grade. Abbreviations used for particular proteins are mentioned adjacent to the name of each protein. The pI of each protein is mentioned in parentheses.

### Protein concentration determination

The stock solutions of all proteins were prepared in neutral buffer. Protein concentration was determined with BCA (Bicinchoinc acid) kit as well as UV-Visible spectrophotometer (Perkin Elmer Lambda25). Concentration of each protein was taken to be 5 µM.

### pH determination

pH measurements were made using Mettler Toledo Seven Easy pH meter (model S20) serial no-1229505169, which is routinely calibrated by standard buffer. The pH of the buffer for each protein was set two units below and above its isoelectric points. Experiments were conducted in 20 mM of following buffers- pH 1.4 (KCl-HCl); pH 2.4, 2.5, 2.7, 2.94, 3.1, 3.2, (Glycine-HCl); pH 3.4, 3.65, 3.7, 3.9, 4.26, 4.69, 5.1, 5.4, 5.69, 5.8 (Sodium acetate); pH 6.4, 6.5, 6.7, 6.94, 7.1, 7.2, 7.4, 7.6, 7.7, 7.9, 8.4 (MOPS); pH 8.69, 9.1, 9.2, 9.69, 9.8 (Glycine-NaOH) and 12.4, 13.2 (KCl-NaoH). All buffers were filtered through 0.45 µM syringe filters.

To avoid any complication due to micelle formation, the molar concentration of SDS was taken as 500 µM (0.5 mM) which is far less than its critical miceller concentration (CMC) in distilled water (8 mM). Before all spectrophotometric measurements, each protein was incubated in buffer containing 100-fold molar excess of SDS for 1 hour at 25°C. The proteins at neutral pH as well as in buffer at pH below two units of pI, both devoid of SDS, served as control A and control B respectively. Similarly, SDS containing protein samples in the buffer at pH above two units of pI were taken as control C.

### Rayleigh Scattering measurements

Rayleigh scattering measurements were performed on a Hitachi F-4500 fluorescence spectrophotometer at 25°C in a 1 cm path-length cell. Protein samples were excited at 350 nm and spectra were recorded in the range of 300–400 nm. Both excitation and emission slits were fixed at 5 nm. Data were plotted at 350 nm.

### Turbidity measurements

The turbidity of the all protein samples below two units of pI in the presence and absence of SDS was monitored by UV absorbance at 350 nm using a Perkin Elmer UV/VIS spectrometer model lambda 25 in a cuvette of 1 cm path length. The measurements were carried out at 25°C.

### ThT binding assay

A stock solution of Thioflavin T (ThT) was prepared in double distilled water. The concentration of ThT was determined using extinction coefficient of 36000 M^−1^ cm^−1^. Protein samples at different pH values were incubated in 1∶1 molar ratio of ThT for 30 minutes at 25°C. The fluorescence of ThT was excited at 440 nm. The spectra was recorded from 450 nm to 600 nm and plotted at 485 nm. The excitation and emission slit width were both fixed at 10 nm. Spectra were also subtracted from appropriate blanks.

### Circular dichroic (CD) measurements

Circular dichroic measurements were performed on a JASCO spectropolarimeter (J-815). The instrument was calibrated with D-10-camphorsulfonic acid. All measurements were made at 25°C with a thermostatically controlled cell holder attached to a peltier with Multitech water circulator. Spectra were collected with a scan speed of 100 nm/min and a response time of 2 s. Each spectrum was average of three scans. Far-UV CD spectra were taken in the range of 200–250 nm range in a cell of 0.1 cm path length. All spectra were smoothed by the Savitzky–Golay method with 25 convolution width. The percent secondary structure content for alpha class proteins was calculated by K_2_D method.

### Transmission Electron Microscopy (TEM)

Transmission electron micrographs were collected on JEOL transmission electron microscope operating at an accelerating voltage of 200 kV. Fibril formation was assessed by applying 6 µl of protein sample (5 µM) on 200-mesh copper grid (CF 200-Cu, lot no-110323) covered by carbon-stabilized Formvar film. Excess of fluid was removed after 2 min and the grids were then negatively stained with 2% (w/v) uranyl acetate. Images were viewed at 10000×.

## Results

### Rayleigh Scattering and Turbidity measurements

**Figure 1:**
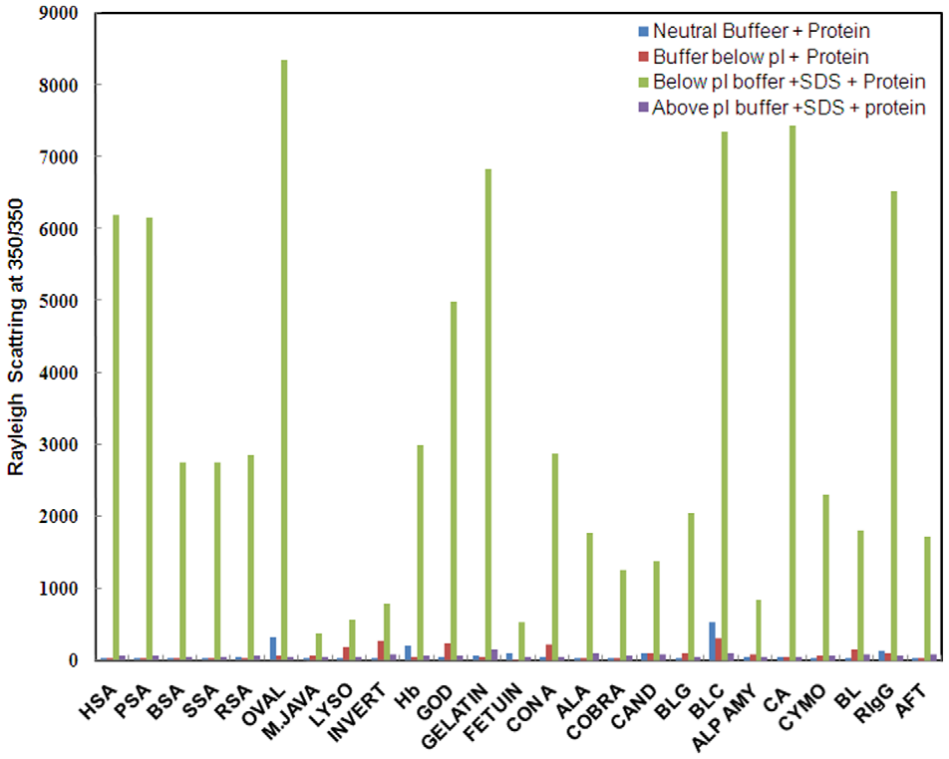
Rayleigh scattering measurements at 350 nm in the absence as well as presence of SDS at pH below and above two units of respective pI. Data at neutral pH+protein [dark blue], pH below pI+protein [red], pH below pI+SDS+protein [light green], pH above pI+SDS+protein [purple]. The molar ratio of protein to SDS was 1∶100.

**Figure 2:**
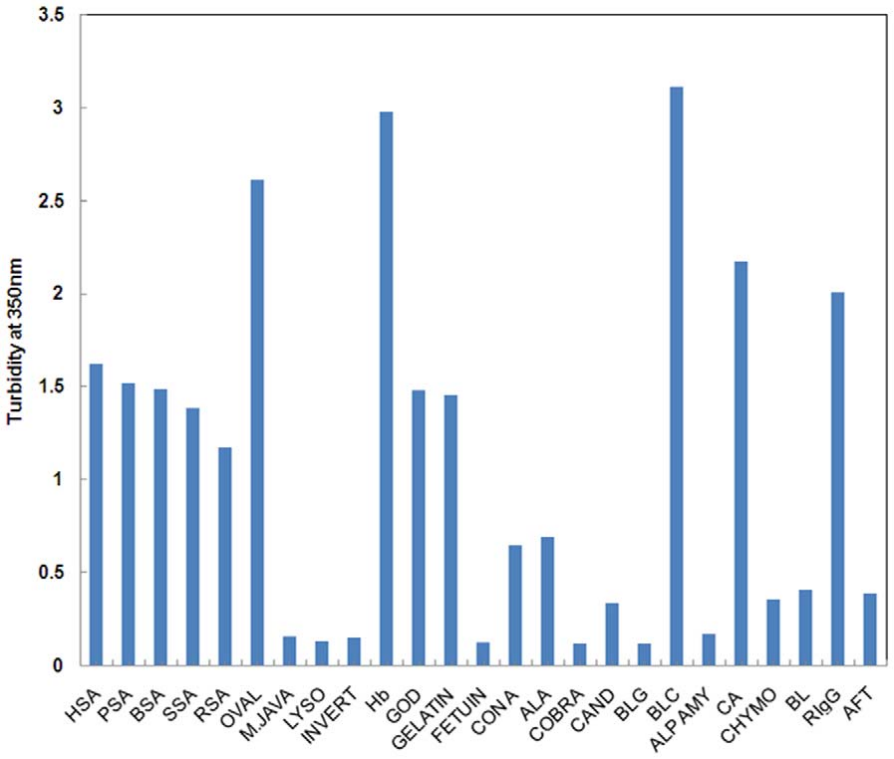
The turbidity measurement of proteins at: 350 nm below two units of pI in the presence of SDS.

Fluorescence intensity (F.I) at 350 nm is a sensitive probe to detect aggregation in proteins. The extent of light scattering in protein samples, below two units of pI, was measured in absence and presence of SDS. A marked increase in fluorescence intensity at 350 nm was observed in the samples incubated with SDS in buffer at pH below two units of pI (Fig. 1). The increase in F.I. was more than five-fold for all proteins taken in the present study as compared to controls A, B and C suggesting the aggregate formation (Table 1) [9]. The possibility of pH dependent aggregation in proteins was ruled out by measuring the scattering at neutral pH (control A) as well as at pH below two unit of pI (controls B). As can be observed from figure, only a slight increase in F.I. occurred in control B as compared to control A, implying that the impact of pH on aggregation appears to be far less than that of SDS. The turbidity measurements taken at 350 nm for protein incubated with SDS at pH below pI, also revealed significantly enhanced turbidity, thus giving further evidence in support of aggregate formation (Fig. 2 and Table 1).

**Table 1:** Spectroscopic properties of all proteins under various experimental conditions.

	Rayleigh Scattering at 350 nm				Turbidity at 350 nm			ThT fluorescence intensity at 485 nm			
Protein	pI	pH below pI+protein	pH below pI+SDS+protein	pH above pI+SDS+protein	pH below pI+protein	pH below pI+SDS+protein	pH above pI+SDS+protein	pH below pI+protein	pH below pI+SDS+protein	pH below pI+SDS	pH above pI+SDS+protein
Human Serum Albumin	5.67	36.01	6191	61.22	0.01	1.62	0.01	2.17	39.80	4.24	3.12
Porcine Serum Albumin	5.89	27.38	6160	61.00	0.01	1.52	0.01	2.00	158.50	4.56	4.54
Bovine Serum Albumin	5.65	28.88	2754	59.00	0.01	1.49	0.01	2.02	32.90	3.84	3.99
Sheep Serum Albumin	5.63	30.10	2750	60.00	0.01	1.39	0.02	2.07	218.40	4.35	4.08
Rabbit Serum Albumin	5.70	42.45	2860	72.00	0.01	1.18	0.01	2.43	171.20	3.60	3.68
Ovalbumin	4.50	68.47	8341	47.75	0.01	2.61	0.01	2.76	52.97	3.92	4.30
*M. javanicus* lipase	5.90	75.86	386	45.00	0.01	0.16	0.01	2.04	16.11	4.42	5.88
Lysozyme	11.20	189.10	561	53.36	0.01	0.14	0.01	4.20	83.97	4.85	6.89
Invertase	3.40	268.20	793	78.00	0.01	0.16	0.02	7.33	84.78	3.96	4.79
Hemoglobin	7.10	56.41	2986	69.00	0.02	2.98	0.01	2.65	12.30	4.42	3.46
Glucose oxidase	4.94	249.30	4996	60.60	0.01	1.48	0.01	27.09	165.10	4.19	3.10
Gelatin	4.70	44.82	6828	146.80	0.01	1.45	0.01	1.94	25.38	4.10	3.23
Fetuin	5.21	25.77	532	59.16	0.01	0.13	0.01	1.89	52.75	3.92	3.23
Concanavalin A	5.43	222.60	2871	48.20	0.01	0.65	0.03	3.05	64.68	4.55	2.13
α- lactalbumin	4.40	31.31	1766	98.00	0.01	0.69	0.01	1.82	52.38	3.93	4.12
Cobra toxin	7.69	32.64	1250	74.00	0.01	0.12	0.01	1.98	30.69	4.55	3.98
*C.rugosa* lipase	4.70	101.10	1384	80.00	0.01	0.34	0.01	3.17	22.97	4.08	2.99
β-lacto globulin	5.20	109.40	2056	50.62	0.01	0.12	0.01	2.42	8.71	4.53	3.98
Bovine liver catalase	5.40	311.30	7349	111.00	0.02	3.11	0.01	11.22	22.63	4.80	4.79
α-amylase	5.90	85.83	843	46.86	0.01	0.17	0.01	8.77	28.07	4.35	6.89
Conalbumin	6.69	59.08	7433	51.66	0.02	2.17	0.01	1.96	75.02	4.33	5.44
Chymopapain	10.40	67.00	2309	69.00	0.01	0.36	0.01	5.90	54.04	3.98	4.34
Banana lectin	6.26	152.90	1812	78.00	0.01	0.41	0.01	1.77	44.37	4.49	4.57
Rat IgG	7.80	107.50	6525	63.00	0.01	2.01	0.01	2.42	50.82	4.37	5.46
Asialofetuin	5.10	36.23	1720	90.00	0.01	0.39	0.01	2.61	86.31	4.31	5.79

### ThT binding studies

**Figure 3:**
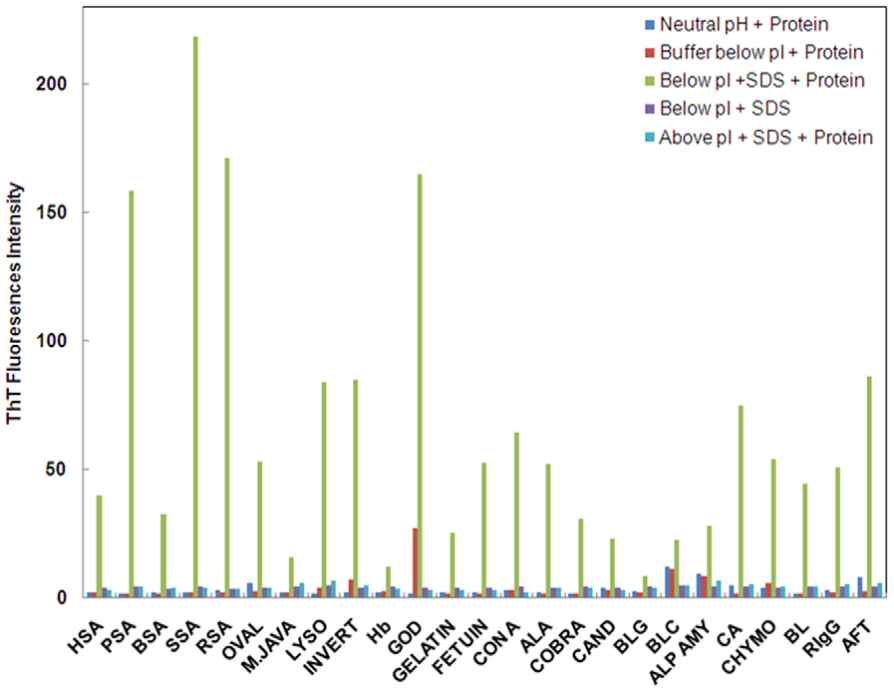
ThT fluorescence intensity data at 485 nm at: neutral pH+protein [dark blue], pH below pI+protein [red], pH below pI+SDS+protein [light green], pH below pI+SDS [purple], pH above pI+SDS+protein [light blue] The molar ratio of protein to ThT was 1∶1.

ThT is a benzothiazole dye that exhibits enhanced fluorescence emissions upon binding to amyloid fibrils and does not bind to amorphous aggregates as well as non amyloid structures [10]. Thus, ThT binding assay was performed to characterize the nature of aggregates induced by SDS. Protein samples containing SDS at pH below two units of pI revealed strong ThT binding as indicated by enhanced ThT fluorescence intensity whereas little or no change in ThT fluorescence was noticed in controls A, B and C (Fig. 3 and Table 1). The results confirm the formation of aggregates with fibrillar structure and thus, the possibility of amorphous aggregate formation was ruled out.

### Secondary Structure Determination

**Figure 4:**
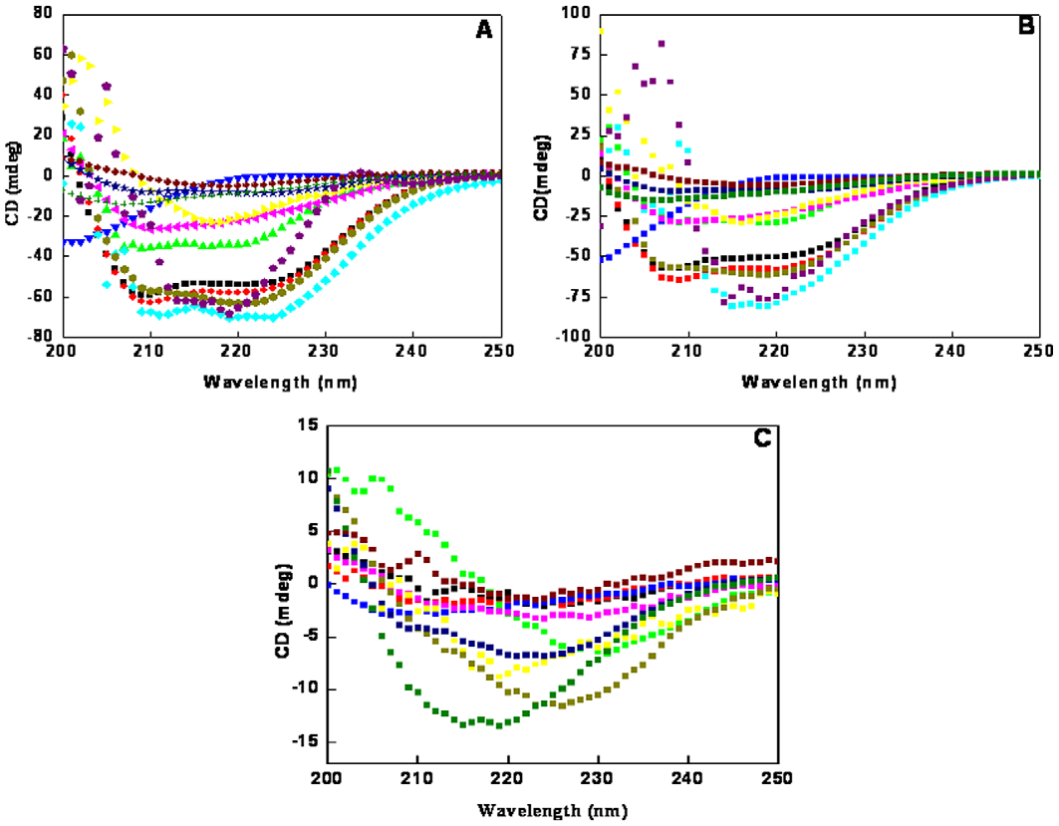
Far-UV CD spectra of the proteins at: [A] neutral pH, [B] pH below pI in absence of SDS, and [C] pH below pI in presence of SDS. Spectra are shown for HSA [black], RSA [red], GOD [green], Gelatin [blue], BLC [aqua], CA [pink], RIgG [yellow], Oval [dark green], Hb [dark blue], Invert [purple], BL [dark red], AFT [orange]. Some Spectra are omitted for sake of clarity. [The presecent of secondry sturucture contents for α-class protein was calculated by K2D methods].

Far UV-CD was used to determine the secondary structure content of proteins. On the basis of secondary structure, several classes of proteins were used in the present study including α-class, β-class, α+β class and proteins with pronounced random coil conformation. α-class and α+β class proteins exhibit two negative peaks, one at 208 nm and other at 222 nm, while β-class proteins show single negative peak between 215 nm and 222 nm. Random coil proteins give single negative peak around 200 nm. The influence of SDS induced aggregates on secondary structure contents of all proteins remained unaltered in control samples A and B (Figs. 4A and 4B). In the presences of SDS, however, pronounced conformational changes were observed (Fig. 4C). All α-class proteins acquired a single negative peak suggesting their transition into β-sheet conformation while proteins belonging to α+β class showed a gain in their β-sheet content. Similarly in β-class and random coil proteins, the negative elipticity reduced upon aggregation. The above secondary structure transitions are characteristic features of amyloid fibrils [11]–[13]. Table 2 summarizes the secondary structure content for only α-class proteins since K_2_D software is not sensitive enough for other classes of protein.

**Table 2:** % Secondary structures content of the proteins in different experimental conditions.

	pH 7.4			pH below pI			pH below pI+SDS		
Protein	α-helix	β-sheet	RC	α-helix	β-sheet	RC	α-helix	β-sheet	RC
Human serum albumin	60	8	32	57	10	33	5	47	48
Porcine serum albumin	62	7	31	59	8	33	5	47	48
Bovine serum albumin	62	7	31	60	8	32	5	47	48
Sheep serum albumin	62	5	33	59	8	33	5	47	48
Rabbit serum albumin	63	6	31	64	6	30	4	48	48
Ovalbumin	80	13	7	84	8	8	4	48	48
Lysozyme	41	15	44	43	22	35	4	48	48
Hemoglobin	60	5	35	61	5	34	4	44	48
Glucose oxidase	38	3	59	29	16	55	9	47	44
Conalbumin	36	21	53	27	19	54	5	47	48
Chymopapain	29	16	55	30	14	56	17	32	51
Bovine liver catalase	27	18	55	25	27	48	16	39	45
α-lactalbumin	25	24	51	17	34	49	12	41	47
Asialofetuin	12	40	48	19	31	50	18	38	54

### Morphology determination by TEM

**Figure 5:**
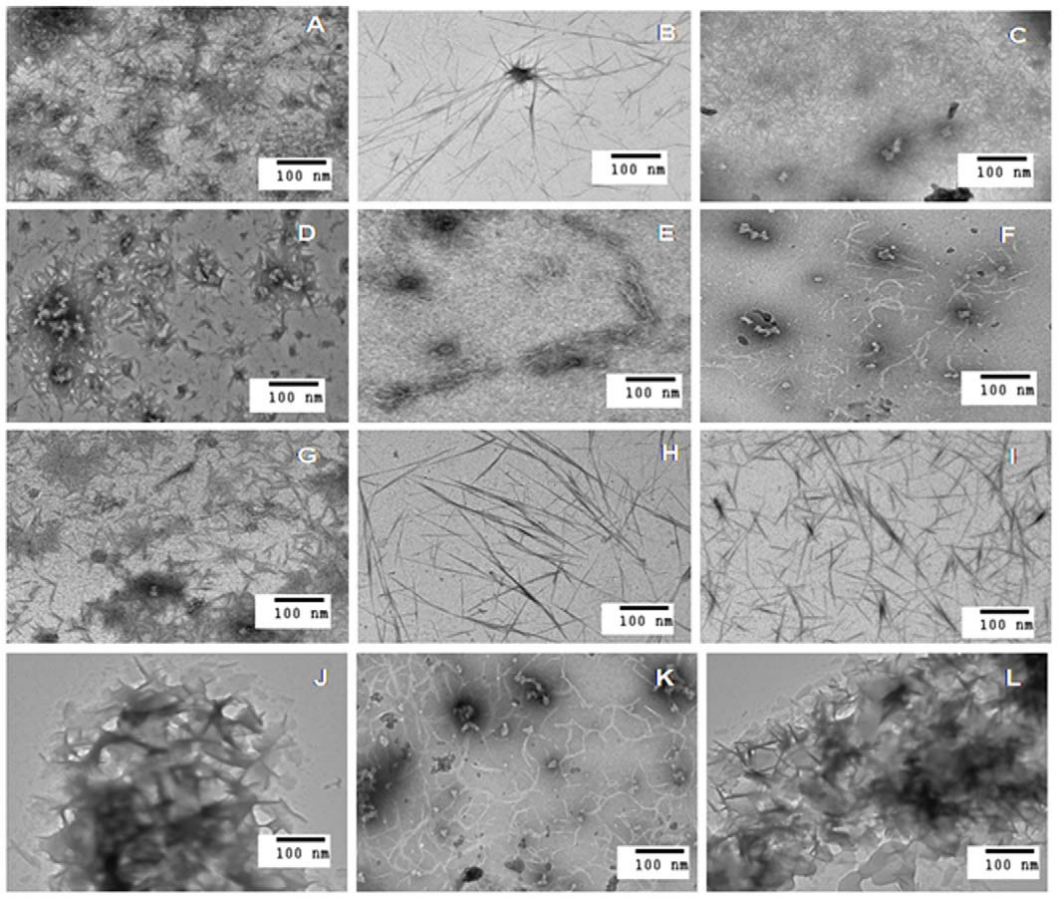
TEM images of 12 representative proteins showing amyloid fibrils in the presence of SDS below two unit of their pI. [A] AFT, [B] ALA, [C] BLC, [D] CA, [E] Cand, [F] Con A, [G] Gelatin, [H] GOD, [I] Hb, [J] HSA, [K] Oval, [L] RSA.

The morphology of aggregates formed was further investigated using electron microscopy. In past, several authors have reported amyloid induction in the presence of SDS [14]–[15]. Proteins in respective below pI buffers were incubated with 100-fold molar excess of SDS for 1 hr and were subsequently examined. Fibriller structures resembling typical amyloid fibril were clearly noticeable in all protein samples (Fig. 5). However, no fibril formation was detected in the samples without SDS (data not shown).

## Discussion

**Figure 6:**
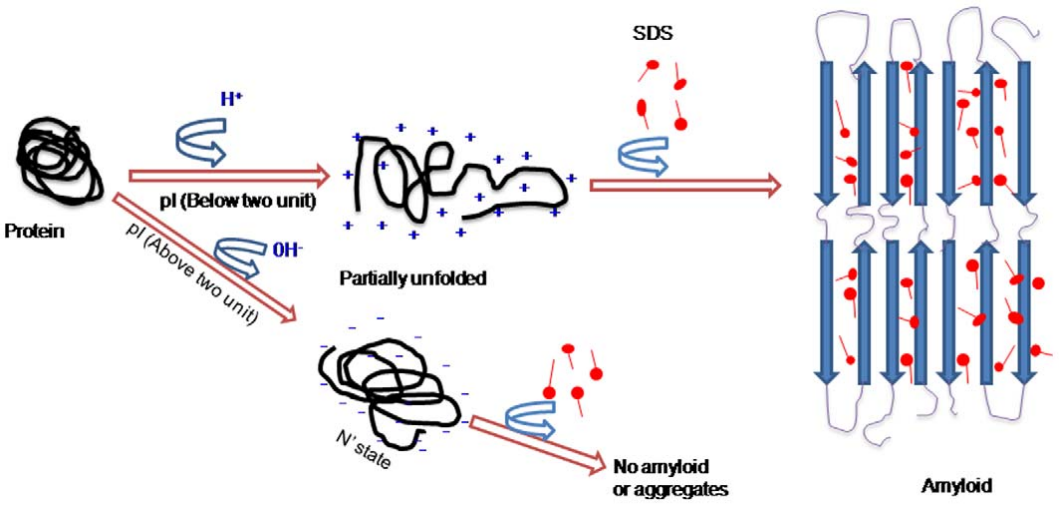
A hypothetical model for amyloid formation.

The aggregation of proteins in the presence of very low concentration of SDS has already been reported earlier but the role of pI in SDS induced aggregation has not yet been discussed [16]–[19]. The partially unfolded state of a protein produced under high temperature, high pressure, low pH and moderate concentration of organic solvents exhibits a tendency towards aggregation [20]–[22]. In the present study we have made an attempt to devise a general approach to induce aggregation in proteins and explore the mechanism behind aggregation by anionic surfactant. It is well documented that proteins acquire net positive charge below its isoelectric point. This results in electrostatic repulsion among charged moieties, leading to partial unfolding of the molecules and thus exposure of hydrophobic patches. A convenient strategy to induce aggregation in such system requires neutralization of positive charge along with increased hydrophobic interactions. Both of these requirements can be successfully satisfied by an amphipathic anionic detergent such as sodium dodecyl sulphate (SDS). SDS contains negatively charged polar head groups and a twelve carbon long non-polar hydrophobic tail. In general, the negatively charged head group of SDS interacts with positive charge developed on protein and the hydrophobic region makes contact with its protein counterpart by repelling the water molecules wrapped around the protein molecule. As a result, solute-solvent interactions are perturbed leading to the induction of aggregates [23]. In the present study, a total of 25 proteins belonging to different families and conformational classes were subjected to pH below two units of their respective pI followed by incubation with 100-fold molar excess of SDS. Aggregation occurred readily in all proteins possibly due to neutralization of the positive charge (developed below pI) and provision of favorable hydrophobic interaction by SDS, thus collapsing the molecules into aggregates. Furthermore, to investigate the leading forces behind the aggregation phenomenon, we conducted the same experiment at pH above two units of pI in the presences of SDS. We, however, could not get aggregates under this condition possibly due to the development of net negative charge (above pI) which did not allow the interaction of negatively charged SDS head groups with the proteins because of charge-charge repulsion. These findings suggest the possible role of electrostatics interactions behind amyloid induction. Besides, the involvement of electrostatic interactions in aggregation is also highlighted by the fact that the negatively charged group present in polysaccharide chain of heparin and heparin sulphate of GAG enhances the aggregate formation in mAcP [24]. Similar findings have been reported by group of Shweta et al [25], where in the presence of anion, moPrP molecules becomes a worm-like fibrils because anion binds to opposite-charged proteins via neutralizing charge-charge repulsion. Studies have reported that elimination of the terminal charges of tetrapeptide KFFE and KVVE by acetylating the N-terminus and amidating the C-terminus appreciably reduced fibril formation. Similarly, fibril formation of KFFE and KVVE was more prominent in water than in phosphate buffer, demonstrating that charge attractions are important in fibril formation [26]. These findings suggest that electrostatic interactions play a major role in aggregation. The twelve carbon- long chain of SDS, however, is large enough to avoid the importance of hydrophobic interaction in the aggregation process. This is also proved by the fact that detergent (MMPA) with longer hydrocarbon chain length (12-C) induces aggregation while those (MCPA) with shorter (6-C) acyl chain are incapable in this regard [27]. Furthermore, it has been shown that SDS (anionic) but not DTAC (cationic), SB12 (amphipathic) and TX 100 (non-ionic) surfactants interact with β2 microglobulin and promote extension of fibril reaction [18]. All of these findings revealed that both electrostatic as well as hydrophobic forces are a prerequisite for all aggregation processes but electrostatic interactions plays a leading role. Figure 6 shows a hypothetical model describing the present findings where proteins, when subjected to pH below two units of pI, acquire a net positive charge and become partially unfolded. In the presence of appropriate molar concentration of SDS, opposite charges are neutralized along with the provision of favorable hydrophobic interactions between non polar hydrocharbon tails of SDS and hydrophobic patches of proteins leading to amyloid induction. However above pI the proteins molecules attain net negative charge forming N′ state which exhibits resistance to aggregation towards SDS due to charge-charge repulsion.

In summary, data presented here indicated that the SDS molecules have great potential to induce amyloid formation when proteins are in partially unfolded states (below two unit of pI). Besides we found that for the formation of amyliod fibril both electrostatics as well as hydrophobic interactions are responsible but has former an upper hand. This study is also very helpful for those working in the area of solublization of misfolded proteins since it provides an easier approach to induce the aggregation. Further studies are required to elucidate the type of fibrils formed whether a cross-β, rigid, straight, spherulitic or worm-like structures.
